# Supplementary material for: Non-pharmacological labor pain management practice and associated factors among skilled attendants working in public health facilities in Gamo and Gofa zone, Southern Ethiopia: A cross-sectional study
Source: PLoS One. 2022 Apr 21;17(4):e0266322. doi: 10.1371/journal.pone.0266322 (PMC9022872; doi:10.1371/journal.pone.0266322)
Supplement: Attachment — Submitted filename: PONE-D-20-00957_reviewer.pdf [file pone.0266322.s002.pdf]

PLOS ONE

Non-Pharmacological Labor Pain Management Practice and Associated Factors among Skilled Attendants working in public health facilities in Gamo and Gofa zone. Southern Ethiopia

--Manuscript Draft--

**Manuscript Number:**	PONE-D-20-00957
**Article Type:**	Research Article
**Full Title:**	Non-Pharmacological Labor Pain Management Practice and Associated Factors among Skilled Attendants working in public health facilities in Gamo and Gofa zone. Southern Ethiopia
**Short Title:**	Non-pharmacological Labor Pain Management Practice and Associated Factors
**Corresponding Author:**	biresaw wassihun alemu, Msc<br>Arba Minch University<br>Arba minch, SNNPR ETHIOPIA
**Keywords:**	Non-pharmacological, Labour pain, management, skilled care, Ethiopia
**Abstract:**	Back ground<br><br>Labour pain management is crucial to ensure the quality of obstetric care and meet the need of laboring woman to have pain free labour and delivery services but it is one of the neglected areas in obstetrics. In low resource country like Ethiopia with low institutional delivery managing labour pain can help as an encouragement to increase institutional delivery.<br><br>Objective<br><br>Non-Pharmacological Labor Pain Management Practice and Associated Factors among Skilled Attendants working in public health facilities in Southern, Ethiopia<br><br>Methods<br><br>An institution based cross-sectional study design was employed on 272 obstetrics care providers working in public health facilities of Gamo and Gofa zone from November 15 –December 30, 2018/2019. Interview administered, pre-tested and a semi- structured questionnaire with observational checklist was used to collect the data. Data was checked and entered into Epi data version 3.1 then exported to statically package for social science version 22 for analysis. Univariate, Bivariate and multivariable analysis with 95% CI was carried out<br><br>Result<br><br>The finding of this study showed that 37.5% of the health care providers were good practice of non-pharmacological labor pain management with [95% CI (32-43)]. Respondents with clinical experience of 5 years and above were 7 times more likely performing good practice than respondent with clinical experience of less and equal to two years with [AOR=7.00(95%CI, 1.60-3.05)] and respondents who had favorable attitude towards labour pain management were 2.82 times more likely performing good practice than others with [AOR=2.82(95%CI, 1.56,5.07)]. In addition to this those health care providers who were working in favorable delivery rooms were 3.45 times more likely perform good practice than others with [AOR= 3.45(95%CI, 2.09, 7.43)]<br><br>Conclusion<br><br>This study revealed poor practice of non -pharmacological labour pain management. Favorable attitude, higher clinical years of experience, midwife in profession and having favorable delivery room were found to be significant predictor of practice of non -pharmacological labour pain management. So, there is a need to shape attitude of skilled attendants towards labour pain management and crating favorable delivery room and raise the awareness of health care providers to use pain-relief methods as

Powered by Editorial Manager® and ProduXion Manager® from Aries Systems Corporation

	part of improving the quality of care during labor and delivery is vital
Order of Authors:	biresaw wassihun alemu, Msc
	Yosef Alemayehu
Additional Information:	
**Question**	**Response**
**Financial Disclosure**<br><br>Enter a financial disclosure statement that describes the sources of funding for the work included in this submission. Review the submission guidelines for detailed requirements. View published research articles from *PLOS ONE* for specific examples.<br><br>This statement is required for submission and **will appear in the published article** if the submission is accepted. Please make sure it is accurate.<br><br>**Unfunded studies**<br>Enter: *The author(s) received no specific funding for this work.*<br><br>**Funded studies**<br>Enter a statement with the following details:<br>• Initials of the authors who received each award<br>• Grant numbers awarded to each author<br>• The full name of each funder<br>• URL of each funder website<br>• Did the sponsors or funders play any role in the study design, data collection and analysis, decision to publish, or preparation of the manuscript?<br>• **NO** - Include this sentence at the end of your statement: *The funders had no role in study design, data collection and analysis, decision to publish, or preparation of the manuscript.*<br>• **YES** - Specify the role(s) played.<br><br>* typeset	The author(s) received no specific funding for this work
**Competing Interests**<br><br>Use the instructions below to enter a competing interest statement for this submission. On behalf of all authors, disclose any competing interests that could be perceived to bias this	The authors have declared that no competing interests exist

work—acknowledging all financial support and any other relevant financial or non-financial competing interests.<br><br>This statement **will appear in the published article** if the submission is accepted. Please make sure it is accurate. View published research articles from *PLOS ONE* for specific examples.<br><br>**NO authors have competing interests**<br><br>Enter: *The authors have declared that no competing interests exist.*<br><br>**Authors with competing interests**<br><br>Enter competing interest details beginning with this statement:<br><br>*I have read the journal's policy and the authors of this manuscript have the following competing interests: [insert competing interests here]*<br><br>* typeset	
**Ethics Statement**<br><br>Enter an ethics statement for this submission. This statement is required if the study involved:<br><br>• Human participants<br>• Human specimens or tissue<br>• Vertebrate animals or cephalopods<br>• Vertebrate embryos or tissues<br>• Field research<br><br>Write "N/A" if the submission does not require an ethics statement.<br><br>General guidance is provided below. Consult the submission guidelines for detailed instructions. **Make sure that all information entered here is included in the Methods section of the manuscript.**	The study protocol was ethically approved by the Ethical Review Board (IRB) of Arba Minch University College of Medicine and Health Sciences. Official letter was written to each hospital and health center. The study posed a low or not more than a minimal risk to the study participants. Also, the study did not involve any invasive procedures. Accordingly, after the objective of the study was explained, verbal informed consent was obtained from all participants

Powered by Editorial Manager® and ProduXion Manager® from Aries Systems Corporation

**Format for specific study types**<br><br>**Human Subject Research (involving human participants and/or tissue)**<br>• Give the name of the institutional review board or ethics committee that approved the study<br>• Include the approval number and/or a statement indicating approval of this research<br>• Indicate the form of consent obtained (written/oral) or the reason that consent was not obtained (e.g. the data were analyzed anonymously)<br><br>**Animal Research (involving vertebrate animals, embryos or tissues)**<br>• Provide the name of the Institutional Animal Care and Use Committee (IACUC) or other relevant ethics board that reviewed the study protocol, and indicate whether they approved this research or granted a formal waiver of ethical approval<br>• Include an approval number if one was obtained<br>• If the study involved *non-human primates*, add *additional details* about animal welfare and steps taken to ameliorate suffering<br>• If anesthesia, euthanasia, or any kind of animal sacrifice is part of the study, include briefly which substances and/or methods were applied<br><br>**Field Research**<br><br>Include the following details if this study involves the collection of plant, animal, or other materials from a natural setting:<br>• Field permit number<br>• Name of the institution or relevant body that granted permission	
**Data Availability**<br><br>Authors are required to make all data underlying the findings described fully available, without restriction, and from the time of publication. PLOS allows rare exceptions to address legal and ethical concerns. See the PLOS Data Policy and FAQ for detailed information.	No - some restrictions will apply

A Data Availability Statement describing where the data can be found is required at submission. Your answers to this question constitute the Data Availability Statement and **will be published in the article**, if accepted.<br><br>**Important:** Stating 'data available on request from the author' is not sufficient. If your data are only available upon request, select 'No' for the first question and explain your exceptional situation in the text box.<br><br>Do the authors confirm that all data underlying the findings described in their manuscript are fully available without restriction?	
**Describe where the data may be found in full sentences. If you are copying our sample text, replace any instances of XXX with the appropriate details.**<br><br>• If the data are **held or will be held in a public repository**, include URLs, accession numbers or DOIs. If this information will only be available after acceptance, indicate this by ticking the box below. For example: *All XXX files are available from the XXX database (accession number(s) XXX, XXX.).*<br>• If the data are all contained **within the manuscript and/or Supporting Information files**, enter the following: *All relevant data are within the manuscript and its Supporting Information files.*<br>• If neither of these applies but you are able to provide **details of access elsewhere**, with or without limitations, please do so. For example:<br><br>*Data cannot be shared publicly because of [XXX]. Data are available from the XXX Institutional Data Access / Ethics Committee (contact via XXX) for researchers who meet the criteria for access to confidential data.*<br><br>*The data underlying the results presented in the study are available from (include the name of the third party*	authors have permission to share the data.

Powered by Editorial Manager® and ProduXion Manager® from Aries Systems Corporation

and contact information or URL).<br>• This text is appropriate if the data are owned by a third party and authors do not have permission to share the data.<br><br>* typeset	
Additional data availability information:	Tick here if your circumstances are not covered by the questions above and you need the journal’s help to make your data available.

Powered by Editorial Manager® and ProduXion Manager® from Aries Systems Corporation

Manuscript

Click here to access/download;Manuscript;New Clean manscuriput biresaw.docx

Non-pharmacological labor pain management practice and associated factors among skilled attendants working in public health facilities of Southern Ethiopia: A cross sectional study

Biresaw Wassihun [1*] Yosef Alemayehu[2]

[1,2]Arba Minch university, college of medicine and health science, department of midwifery, Arba Minch, Ethiopia

Email Address

BW= bireswas@gmail.com

YA= jmtumu@gmail.com

corresponding author

*bireswas@gmail.com

Abstract

Background: The health and well-being of a mother and child at birth largely determine the future health and wellness of the entire family. labor pain management is crucial to ensure the quality of obstetric care but it is one of the neglected areas in obstetrics, Therefore the aim of this study was to assess the practice of labor pain management and Associated factors among skilled attendants working in public health facilities in Southern, Ethiopia

Methods: Cross-sectional study design was used among randomly selected 272 obstetrics care providers working at the labor ward. Data were collected using a pretested and structured questionnaire. ~~The collected data were entered into the Epi Data software, version 3.1 and exported~~ to the Statistical Package for Social Sciences (SPSS) software, version 22.0 ~~for~~ analysis. Descriptive statistics were used to describe each variable and logistic regression was used to determine the association between the predictor variables and outcome variables. With 95% confidence interval (CI) and $P < 0.05$ was considered a significant variable

Result: Overall, 37.5% (95%CI: 32, 43%) of health professionals had a good practice of non pharmacological labor pain management. Clinical experience of 5 years and above [AOR=7.00(95%CI, 1.60-3.05)], favorable attitude [AOR=2.82(95%CI, 1.56,5.07)], midwife profession [AOR=7.13(95% CI,1.06-4.77)] and working in favorable delivery rooms [AOR= 3.45(95%CI, 2.09, 7.43)] were significantly associated with a health professionals good practice of labor pain management

Conclusion: This study found that the practice of non-pharmacological labor pain management was poor. It was observed that; having a favorable attitude, increased clinical years of experience, being a midwife by professional and having favorable delivery room were found to be a significant predictor of the practice of non -pharmacological labor pain management. So, there is a need to shape the attitude of skilled attendants towards labor pain management and crating favorable delivery room and raise the awareness of health care providers to use non-pharmacological pain-relief methods as part of improving the quality of care during labor and delivery is vital

Keywords: Non-pharmacological, Labour pain, management, skilled care, Ethiopia

Background

Women's experience of pain during labor varies greatly. Some women feel little pain whilst others find the pain extremely distressing. A woman's position in labor, mobility, and fear and anxiety or, conversely, confidence may influence her experience of pain (1,2). ~~Irrespective of the grades and strength of their pain, most laboring women need pain relief and require various pharmacological and non-pharmacological pain relief interventions (3).~~ Effective management of labor pain plays a pivotal role in a woman's satisfaction with childbirth (4-6). Its practice varies from one clinical setup to another. In a developed country pharmacologic way of pain relief is the preferred one (7).

Globally maternal mortality is unacceptably high. About 295 000 women died during and following pregnancy and childbirth in 2017. The vast majority of these deaths (94%) occurred in low-resource settings, and most could have been prevented. Sub-Saharan Africa and Southern Asia accounted for approximately 86% (254 000) of the estimated global maternal deaths in 2017(8). According to EDHS 2016 report MMR in Ethiopia was 412 per 100,000 live birth. Only 26% of births in Ethiopia were delivered at a health facility by a skilled attendant (9). Lack of labor pain management practice is one of the unspoken causes of low utilization of institutional delivery (9). Studies have been shown that when women are offered analgesia during labor, they report greater satisfaction with their overall birth experience and facilitate mothers' ability for attachment and infant car giving (10-11). Providing pain relief during labor and delivery has many advantages like having shorting the progress of labors, minimizing the need for oxytocin and anesthesia, the strength of maternity care user and providers bonding, reducing instrumental deliveries, and decreased by 50% of cesarean section(12). Additionally, Anxiety, postpartum-depression, postpartum bleeding, postpartum fever, a low Apgar score of neonates (<7 at 5 minutes) and prolonged infant hospitalization can be also decreased (13). The Ethiopian FMoH has developed and implemented the first standard of midwifery care practice in 2013. Among the practice competencies stated; the provision of physical and psychological support and use of pharmacological and non-pharmacological comfort measures during labor and birth are listed as core competencies under Practice Standard III(14). This is one of the components to improve the quality of maternal health services. However, its actual practice is not clearly recognized. (15,16). Therefore, this study has assessed the practice of labor pain management and associated factors

among skilled attendants working in public health facilities in Gamo and Gofa zone. Southern Ethiopia

Methods

Study area and period

Gamo and Gofa are one of the 15 zones in Southern Nations, Nationalities and Peoples Region (SNNPR). The administrative center of Gamo and Gofa is Arba Minch. Based on the 2007 Census conducted by the Central Statistical Agency of Ethiopia, this Zone has a total population of 1,593,104, of whom 793,322 are men and 799,782 women; with an area of 18,010.99 square kilometers. The study was conducted in selected public health facilities in Gamo and Gofa zone in south Ethiopia, from September 1 to December 26, 2018

Study design

The facility-based cross-sectional study design was employed

Study population

All skilled obstetric caregivers who were working at the labor ward in public health institution of Gamo and Gofa zone

Sample size determination

The sample size was determined by using Epi Info 7 menu Stat Calc, by considering the following assumptions: confidence level 95%, power 80% and exposed to the unexposed ratio of 1:1 (**Table 1**)

Table 1. Sample Size Calculation to assess the practice of labor pain Management in Gamo and Gofa zone Public health facility southern, Ethiopia

**Factors associated with the practice of labor pain**	**Variables**	**The practice of labor pain Management among exposed and non-exposed**	**Sample size**
	Knowledge level	**19.8%** those health care providers who have Good knowledge	**256**
		**20.3%** those health care providers who have poor knowledge	
	Profession	35% of those health care providers who are midwives	**234**

		18% of those health care providers who are not a midwife	
	Educational level	**40.9%** those health care providers having a diploma	**84**
		**81% of** health care providers having a degree and above	

Finally, the required sample size for this particular study was decided by taking the maximum sample size from the calculation results. Thus, **256** health care providers were included in this study by adding a 10% non-response rate the final sample size was 282

Operational definition

Good knowledge: Those skilled health care providers /respondents who scored equal and above mean score of the knowledge questions.

Poor knowledge: Those skilled health care providers /respondents who scored below mean score of the knowledge questions.

Good practice: Those health care providers /respondents who scored equal and above mean score of the labor pain management practice

Poor practice: Those health care providers /respondents who scored below mean score of the practice questions

Data collection tool and sampling procedure

A structured and pretested interviewer-administered questionnaire with an observational cheek list was used. The questionnaire was designed in English and it was translated to the Amharic language by the translator, and then translated back to English by a third person to check for consistency. All skilled attendants who were working in labor wards of public health institutions found in the Gamo and Gofa zone and who fulfilled eligibility criteria were interviewed by structured questionnaires. Nine data collectors were recruited principal investigators supervise the data collection process

Data quality control

To maintain the validity of data to be collected randomly selected study participants were involved and standard questionnaires and checklist were used. The pre-test was conducted for

inconsistencies and misunderstandings of the questions to assure reliability. Extensive training was given to data collectors and supervisors regarding the objective of the study and data collection tool. All data were checked for completeness, accuracy, clarity, and consistency by the principal investigator and by supervisors, on the day of data were collected. Data were checked for completeness before data entry into the software. Proper coding and categorization of data were maintained in the quality of the data to be analyzed.

Data analysis

First, the collected data were checked manually for completeness, and entered into EPI-data version 3.1 and transferred to SPSS version 22 for analysis. Descriptive statistics with percentages were employed. All variables were analyzed in bivariate logistic regression and those variables having P-value less than 0.2 were entered to multivariable logistic regression analyses. In multivariable logistic regression analyses variables with P-value, less than 0.05 were considered as significant. Hosmer–Lemeshow goodness of fit test was used to check the model fitness. Adjusted odds ratio with 95% confidence interval was used to determine the presence and direction of the association between covariates and the outcome variable

Results

Socio‐ demographic characteristics

In this study, 272 health professionals participated, yielding a response rate of 96%. The mean age and standard deviation of study participants were 29.9 ± SD4.89 years old. More than half (57%) of the study participants were females and 155(66.5%) were married. 107(39.3%) of health professionals were orthodox religious followers (**Table 2**).

Table 2. Socio-demographic characteristics of respondent in Gamo and Gofa zone Public health facility southern, Ethiopia

Variables	Frequency	Percentage
**Age**		
21-25	60	22.1
26-30	79	29.0
31-35	98	36.0
>=36	35	12.9
**Marital status**		
Married	181	66.5
Divorced	3	1.1
Single	88	32.4
**Sex**		

Male	117	43.0
Female	155	57.0
**Profession**		
Midwife	173	63.6
Nurse	36	13.2
Public health officer	63	23.2
**Highest qualification**		
Masters	14	5.1
Bachelor degree	154	56.6
Diploma	104	38.2
**Religion**		
Orthodox	107	39.3
Protestant	78	28.7
Muslim	87	32.0
**Ethnicity**		
Gamo	94	34.6
Gofa	80	29.4
Wolayta	11	4.0
Amhara	45	16.5
Oromo	22	8.1
Others	20	7.4
**Work experience**		
1-2	90	33.1
3-4	88	32.4
≥ 5	94	34.6

Knowledge of non-pharmacological labor pain management practice

Of the total respondents, 223(82.0%) stated back massage can used to relieve labor pain and majority 217(79.8%) of health care providers mentioned relaxation/breathing technique used to relieve labor pain (**Table 3).** Of the total respondents, 140(51.5%) of health care providers have good knowledge and 132(48.5%) of health care provider have poor knowledge of labor pain management respectively (**Figure 1)**

Table 3. knowledge of non-pharmacological labor pain management practice in Gamo and Gofa zone Public health facilities southern, Ethiopia

Variables	Frequency	Percentage
**Which pharmacologic method do you know**		
**Systemic**		
Yes	181	66.5
No	92	33.5
**NSAID drugs**		

Yes	186	68.4
No	86	31.6
**Epidural analgesia**		
Yes	175	64.3
No	97	35.7
**Inhalational**		
Yes	190	69.9
No	81	29.8
**Which non-pharmacologic method do you know**		
**Psychotherapy**		
Yes	210	73.9
No	71	26.1
**Allow companion of her choice**		
Yes	211	77.6
No	61	22.4
**Show the patient how to bear down**		
Yes	212	77.9
No	60	22.1
**Relaxation/breathing technique**		
Yes	217	79.8
No	55	20.2
**Massage the back**		
Yes	223	82.0
No	49	18
**Allow the mother to ambulate**		
Yes	218	80.1
No	54	19.9
**Trans cutaneous electrical nerve stimulation**		
Yes	77	28.5
No	195	71.7
**Subcutaneous water injection**		
Yes	158	58.1
No	114	41.9
**Diversional therapy**		
Yes	162	59.6
No	110	40.4
**Hypnosis**		
Yes	81	29.8
No	191	70.2
**Acupuncture**		
Yes	83	30.5
No	189	69.5

Music therapy		
Yes	180	66.2
Yes	92	33.8

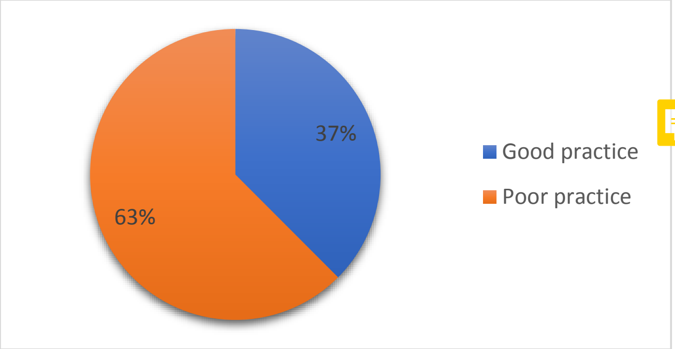

Figure 1, Overall knowledge of health care providers towards non-pharmacological Labour pain management in Gamo and Gofa Zone, Southern Ethiopia

Attitude of non-pharmacological labor pain management practice

Overall, 195(71.7%) of health care providers have unfavorable attitudes of labor pain management and 77(28.3%) of health care providers have a favorable attitude. Majority 263(96.7%) of health care providers believe that analgesia is necessary for managing labor pain and 147(54%) believe that labor pain management reduces postpartum depression. Above the half 197(72%) agree that continuous labor support increase maternal and fetal bonding, in addition, 198(72.8%) of respondents believe that spousal presence during labor facilitate delivery (**Table 4)**

Table 4. The attitude of non-pharmacological labor pain management practice in Gamo and Gofa zone Public health facilities southern, Ethiopia

Variables	Frequency	Percentage
Do you believe labor pain management methods can alleviate or help the mother to cop labor pain		
Yes	205	75.4
No	67	24.6
Do you think every mother pain during labor should be managed		
Yes	263	96.7
No	9	3.3
Do you believe that even though labor pain is natural and mother hasn't to face it?		
Yes	73	26.5

No	199	73.2
Do you think analgesia is necessary for managing labor pain		
Yes	210	73.9
No	71	26.1
Do you believe that labor pain management reduce postpartum depression		
Yes	147	54.0
No	125	46.0
Do you agree that continuous labor support increase maternal and fetal bonding		
Yes	197	72.4
No	75	27.6
Do you believe that spousal presence during labor facilitate delivery		
Yes	198	72.8
No	74	27.2

Practice of non-pharmacological labor pain management

Of the total respondents, majority 154(56.6%) of health care providers allow companions of her choice during labor and delivery and 184(67.6%) health care providers use psychotherapy to relieve labor pain. Majority 182(66.9%) and 210(77.2%) of health care providers use relaxation/ breathing techniques and massage the back to relieve labor pain respectively (**Table 5).** Overall, 170(62.5%) of health care provider have poor practice and 102(37.5%) have good practice of non-pharmacological labor pain management (**Figure 2)**

Table 5. The practice of non-pharmacological labor pain management in Gamo and Gofa zone Public health facility southern, Ethiopia

S.no	List of cheek list	Observational cheek list to asses practice labor pain management	
		**Yes**	**No**
1.	Health Care providers show the patient how to bear down during labor and delivery	153(56.3%)	119(43.7%)
2.	Health Care provider allow companion of her choice during labor and delivery	154(56.6)	118(43.4)
3.	Health care provider massage the back to relieve labor pain	210(77.2)	62(22.8)
4.	Health care providers use hot or cold pack compress to relief labor pain	182(66.9)	90(33.1)

5.	Health care provider use psychotherapy to relief labor pain	184(67.6)	88(32.4)
6.	Health care provider use relaxation/ breathing technique to relieve labor pain	182(66.9)	90(33.1)
7.	Health care provider allows the mother to ambulate/labor exercise	158(58.1)	114(41.9)
8.	Health care provider use Acupuncture to relieve labor pain	32(11.7)	140(88.3)
9.	Health care provider use subcutaneous water injection	112(41.2)	160(58.8)

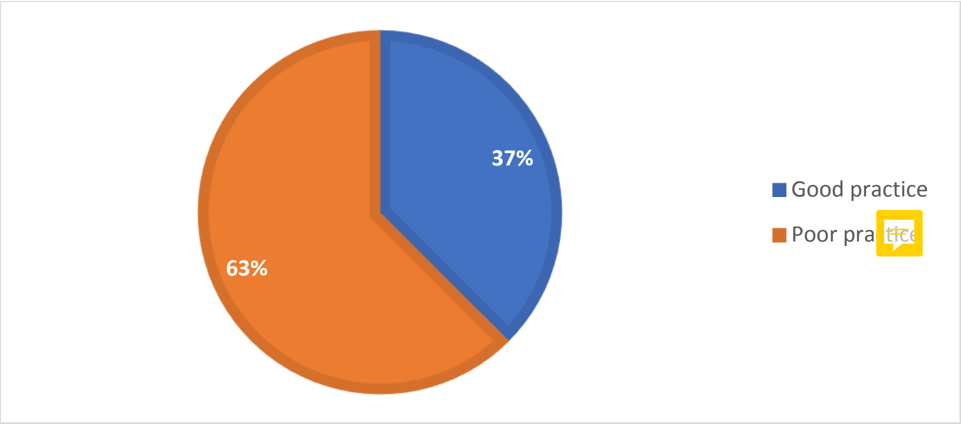

Figure 2, Overall practice of health care providers towards non-pharmacological Labour pain management in Gamo and Gofa Zone, Southern Ethiopia

Factors associated with the practice of non-pharmacological labor pain management

Binary Logistic regression was performed to assess the association of each independent variable with practice labor pain management. The factors that showed a p-value of less than 0.25 were added to the multivariable regression model. The result revealed that on the Bivariate analysis variables like clinical year of experience, sex of health care provider, the attitude of health care providers was some of the variables significantly associated with labor pain management practice In multivariable logistic regression clinical year of experience, having a favorable attitude, having favorable delivery room and being midwifery by profession were some of the variables significantly associated with the practice of non-pharmacological labor pain management at P-value of <0.05.

Respondents with clinical experience of 5 years and above were 7 times more likely performing good practice than respondent with clinical experience of less and equal to two years with

[AOR=7.00(95%CI, 1.60-3.05)] and respondents who had favorable attitude towards labor pain management were 2.82 times more likely performing good practice than others with [AOR=2.82(95%CI, 1.56,5.07)]. In addition to this, those health care providers who were working in favorable delivery rooms were 3.45 times more likely to perform good practice than others with [AOR= 3.45(95%CI, 2.09, 7.43)]. Those health care providers who have midwifery in the profession were 7.13 times more likely perform good practice than others with [AOR= 7.13(95%CI, 1.06, 4.77)] (**Table 6**)

Table 6. Factors associated with the practice of non-pharmacological labor pain management in Gamo and Gofa zone Public health facilities' southern, Ethiopia

**Variables**	**The practice of labor pain management**		**COR with 95% CI**	**AOR with 95% CI**
	**Good practice**	**Poor practice**		
year of experience				
1-2	22	68	1	1
3-4	30	58	1.59(0.83-3.07)	6.98(2.29-12.5) *
≥ 5	50	44	3.51(1,87-6.57) *	7.00(1.60-3.05) *
Knowledge of HCP				
Poor knowledge	54	78	1	1
Good knowledge	48	92	0.75(0.46-1.23)	0.50(0.24-1.16)
Attitude of HCP				
Unfavorable	45	87	1	1
Favorable	32	108	3.40(1.97-5.89) *	2.82(1.56-5.07) **
Gender				
Male	49	68	1	1
Female	53	102	0.72(0.44-1.18)	0.68(0.37-1.23)
Qualification of HCP				
Masters	8	6	1.44(0.46-4.44)	3.55(0.66-19.02)
Bachelor	44	110	0.43(0.26-0.73)	2.14(0.56-8.15)
Degree	50	54	1	1
Profession				

Midwife	71	102	1.39(0.76-2.55)	7.13(1.06-4.77) *
Nurse	10	26	0.76(0.31-1.88)	0.98(0.32-2.92)
Health officer	21	42	1	1
Delivery room status				
Conducive	92	112	0.73 (0.36- 1.25)	3.45(2.09-7.43) *
Not conducive	36	32	1	1

Discussion

~~There is no hesitation that the provision of pain relief services during labor is very important to attract more mothers to health facilities.~~ Pain is more than just a feeling of discomfort lead to mental health conditions like depression and anxiety. This institutional-based cross-sectional study has attempted to the assessed practice of labor pain management methods and associated factors by skilled attendants working in the labor ward, in southern, Ethiopia

In this study, 37.5% (~~95%CI: 32, 43%~~) of the study participants had a good practice of non-pharmacological labor pain management. The magnitude of good practice of health professionals on non-pharmacological labor pain management was in line with a study done in Bangladesh (40%), Kembata Tembaro zone, Southern Ethiopia (37.9%), Amhara region referral hospitals (40.1%), and eastern part of Ethiopia (30.4%) respectively and higher than studies done in Tigray Region, North Ethiopia (43.4%) (1,17,2,18). The reason for this discrepancy in the practice of non-pharmacological labor pain management is because there is an advance in the health care system and refreshment pieces of training are given to health professionals by concerning bodies. The finding of this study is lower than the studies conducted in Nigeria (48.4%) and Benin (48.9%) respectively. The discrepancy might be due to well-organized health facilities, the existence of better education, cultural, and socioeconomic status, which might be the reason to use the labor analgesia to relieve labor pain (21,22)

In this finding, the most practiced non -pharmacological labor pain management was allowing mothers to move freely, show the mothers how to bear down, allow companion during labor and delivery, massage the back which was comparable with the same studies done in Bangladesh and Ghana (1,19)

In multivariable logistic regression clinical year of experience, having a favorable attitude, having favorable delivery room and being midwife by profession were some of the variables significantly associated with the practice of non-pharmacological labor pain management. This finding is in line with another study conducted in Ethiopia(17)

Respondents with clinical experience of 5 years and above were 7 times more likely performing good practice than respondents with clinical experience of less and equal to two years. This result was consistent with the study conducted in northern Ethiopia, this could be the reason that the more they stay in work the more they can understand labor pain. Respondents who had favorable attitudes towards labor pain management were 2.82 times more likely performing good practice than others. This finding is similar to other studies conducted in the northern part of Ethiopia (17). The fact for this is a good attitude of health care professionals is more likely to have the skills and perform the activities in a good manner as much as possible. In addition, those health care providers who were working in favorable delivery rooms were 3.45 times more likely to perform good practice than others. Qualification of the respondents was not significantly associated with this study, but it was significant in other studies

Conclusion

This study revealed the poor practice of non -pharmacological labor pain management. Favorable attitude, higher clinical years of experience, a midwife by profession and having favorable delivery room were found to be significant predictors of the practice of non -pharmacological labor pain management. The identified factors should be addressed to improve pain management for expectant mothers So, there is a need to shape attitude of skilled attendants towards labor pain management and crating favorable delivery room and raise the awareness of health care providers to use pain-relief methods as part of improving the quality of care during labor and delivery is vital. Future prospective studies should be needed to examine the effect of non-pharmacologic labor pain management methods on labor pain relief and other related maternal and neonatal outcome

Declarations

Ethical approval and consent to participant

The study protocol was ethically approved by the Ethical Review Board (IRB) of Arba Minch University College of Medicine and Health Sciences. An official letter was written to each hospital

and health center. The study posed a low or no more than minimal risk to the study participants. Also, the study did not involve any invasive procedures. Accordingly, after the objective of the study was explained, verbal informed consent was obtained from all participants

Availability of data and materials

All related data has been presented within the manuscript. The datasets used are available on the hands of the corresponding author on reasonable request.

Consent for publication

Not applicable

Competing interests

The authors declare that no one has competing interests.

Funding

Not applicable

List of Abbreviations

EDHS: Ethiopian Demographic and health surveillance; AOR: Adjusted odds ratio; CI: confidence interval

Acknowledgment

The authors would like to thank data collectors and respondents who participated in this study.

Author's contribution

Conceptualization: Biresaw Wassihun and Yosef Alemayehu

Formal analysis: Biresaw Wassihun and Yosef Alemayehu

Funding acquisition: Biresaw Wassihun and Yosef Alemayehu

Investigation: Biresaw Wassihun and Yosef Alemayehu

Methodology Biresaw Wassihun and Yosef Alemayehu

Project administration: Biresaw Wassihun and Yosef Alemayehu

Resources: Yosef Alemayehu and Biresaw Wassihun

Supervision: Biresaw Wassihun and Yosef Alemayehu

Validation: Biresaw Wassihun and Yosef Alemayehu

Visualization: Yosef Alemayehu and Biresaw Wassihun

Writing-original draft: Biresaw Wassihun and Yosef Alemayehu

Writing – review & editing: Yosef Alemayehu and Biresaw Wassihun

References

1. Tasnim S. Perception about pain relief during normal labor among health care providers conducting delivery. Medicine Today. 2010;22(1):20-3.
2. Sahil E, Yemaneh Y, Alehegn A, Nigussie W, Salahuddin M, Yekoye A, et al. Practice of Labour Pain Management Methods and Associated Factors among Skilled Attendants Working at General Hospitals in Tigray Region, North Ethiopia: Hospital-Based Cross-Sectional Study Design. Health Science Journal. 2017;11(4).
3. Iliadou M. Labour pain and pharmacological pain relief practice points. Health Sci J. 2009;3(4):197-201.
4. Soet JE, Brack GA, DiIorio C. Prevalence and predictors of women's experience of psychological trauma during childbirth. Birth. 2003;30(1):36-46.
5. Bonnet M-P, Prunet C, Baillard C, Kpéa L, Blondel B, Le Ray C. Anesthetic and obstetrical factors associated with the effectiveness of epidural analgesia for labor pain relief: an observational population-based study. Regional anesthesia and pain medicine. 2017;42(1):109-16.
6. Klein MC, Grzybowski S, Harris S, Liston R, Spence A, Le G, et al. Epidural analgesia use as a marker for physician approach to birth: implications for maternal and newborn outcomes. Birth. 2001;28(4):243-8.
7. Mugambe J, Nel M, Hiemstra L, Steinberg W. Knowledge of and attitude towards pain relief during labor of women attending the antenatal clinic of Cecilia Makiwane Hospital, South Africa. South African Family Practice. 2007;49(4):1.
8. World Health Organization. Trends in maternal mortality from 2000 to 2017: estimates by WHO, UNICEF, UNFPA, World Bank Group, and the United Nations Population Division.
9. WHO. Ethiopia Demographic and Health Survey. The DHS Program ICF Rockville, Maryland, USA.

October 2016.

10. Duarte Ortiz G, Navarro-Vargas JR, Eslava-Schmalbach J. Inequity in the healthcare-The outlook for obstetric analgesia. Revista Colombiana de Anestesiología. 2013;41(3):2157.
11. Ogboli-Nwasor E, Adaji S, Bature S, Shittu O. Pain relief in labor: a survey of awareness, attitude, and practice of health care providers in Zaria, Nigeria. Journal of pain research. 2011;4:227.
12. Emelonye A. Spousal Participation in Labor and Delivery in Nigeria. Annals of Medical and Health Sciences Research. 2014;4(4).
13. Sauls DJ. Effects of labor support on mothers, babies, and birth outcomes. Journal of Obstetric, Gynecologic, & Neonatal Nursing. 2002;31(6):733-41.
14. (FMOH) FMoH. Standard of midwifery care practice in Ethiopia. Addis Ababa (Ethiopia). 2013 Aug: 64p (clinical standard; no. 1.
15. Nystedt A, Edvardsson D, Willman A. Epidural analgesia for pain relief in labor and childbirth–a review with a systematic approach. Journal of clinical nursing. 2004;13(4):455-66
16. Howell C, Kidd C, Roberts W, Upton P, Lucking L, Jones P, et al. A randomized controlled trial of epidural compared with non- epidural analgesia in labor. BJOG: An International Journal of Obstetrics & Gynaecology. 2001;108(1):27-33.
17. Geltore TE, Taye A, Kelbore AG. Utilization of obstetric analgesia in labor pain management and associated factors among obstetric care givers in public health facilities of Kembata Tembaro Zone, Southern Ethiopia. Journal of pain research. 2018;11:3089.
18. Anteneh K. Knowledge, attitude and use of labor pain relief methods and associated factors among obstetric care givers at public health centers of east gojam zone, Amhara Region, Ethiopia, 2018 (Doctoral dissertation, Addis Ababa University).
19. Aziato L, Kyei AA, Deku G. Experiences of midwives on pharmacological and non-pharmacological labor pain management in Ghana. Reproductive health. 2017 Dec;14(1):128.
20. Ohaeri B, Olunaike G, Ingwu J. Skilled health attendants' knowledge and practice of pain management during labor in health care facilities in Ibadan, Nigeria.
21. Roets L, Moru MM, Nel M. Lesotho midwives' utilization of nonpharmacological pain management methods during the first of stage labor. Curationis. 2005;28(3):73–777

22. Hasan MS, Alsaadi ZA, Abbas MA, Algoraby JM. Awareness and attitude of pregnant women towards labor analgesia in Babil Province. Medical Journal of Babylon. 2016;13(1):95-104

Click here to access/download
Supporting Information
Track change.docx
