# Supplementary material for: Non-pharmacological labor pain management practice and associated factors among skilled attendants working in public health facilities in Gamo and Gofa zone, Southern Ethiopia: A cross-sectional study
Source: PLoS One. 2022 Apr 21;17(4):e0266322. doi: 10.1371/journal.pone.0266322 (PMC9022872; doi:10.1371/journal.pone.0266322)
Supplement: Attachment — Submitted filename: Response to reviwers biresaw.docx [file pone.0266322.s003.docx]

**Author’s Point-by-Point Response to the Reviewer's and Editors Reports**

**Title**: Non-pharmacological labor pain management practice and associated factors among skilled attendants working in public health facilities of Southern Ethiopia: A cross sectional study

**Corresponding author**: Biresaw Wassihun /[bireswas@gmail.com](mailto:bireswas@gmail.com)

**Authors**

**1. Biresaw Wassihun**

2. Yosef Alemayehu

**Manscurpuit number:** PONE-D-20-00957

**Journal**: **Plos one**

**Article type:** Research article

**Point by point response to Reviewers and Editors**

First of all, the authors would like to thank Plos one Journal editors and the respective reviewers for reviewing our manuscript and providing the necessary comments to be corrected. As per the comments given, we have made corrections point by point to comment. The authors tried to answer all the issues raised by editorial team and reviewers.

**Point by point response to Editor**

**Dear** Nülüfer Erbil, Ph.D, Prof

1. Please ensure that your manuscript meets PLOS ONE's style requirements, including those for file naming.""

**Response:** Thank you very much we had apply journal requirement

2. We note that you have indicated that data from this study are available upon request. PLOS only allows data to be available upon request if there are legal or ethical restrictions on sharing data publicly

a) If there are ethical or legal restrictions on sharing a de-identified data set, please explain them in detail (e.g., data contain potentially identifying or sensitive patient information) and who has imposed them (e.g., an ethics committee). Please also provide contact information for a data access committee, ethics committee, or other institutional body to which data requests may be sent.

b) If there are no restrictions, please upload the minimal anonymized data set necessary to replicate your study findings as either Supporting Information files or to a stable, public repository and provide us with the relevant URLs, DOIs, or accession numbers

**Response**: no restriction on data we can attach as supplementary files.

3. Please amend either the title on the online submission form (via Edit Submission) or the title in the manuscript so that they are identical

**Response:** Non-Pharmacological Labor Pain Management Practice and Associated Factors among Skilled Attendants working in public health facilities in Gamo and Gofa zone, Southern Ethiopia: A cross sectional study: this is the correct title

4. Your ethics statement must appear in the Methods section of your manuscript. If your ethics statement is written in any section besides the Methods, please move it to the Methods section and delete it from any other section. Please also ensure that your ethics statement is included in your manuscript, as the ethics section of your online submission will not be published alongside your manuscript

**Response**: Thank you very much we had moved it to the Methods section

**Point by point response to Reviewer# 1**

**Question 1:** Thank you for your project on these important topics, I have attached my comments in the file, but my big concern about the instrument that you used; no information is given about the instrument how you measured knowledge attitude of providers.

Who develop them, validity, and reliability?
My all comments are in the pdf file.

**Response 1:** We would like to say thank you very much for your invaluable comments and suggestions. We considered and modified and rewrote again based on your constructive issues regarding language, coherence and comprehensibility of the manuscript

**Question 2**: what dose favorable delivery rooms?

**Response 2:** Favorable delivery room means it is satisfactory delivery room having the presence of the entire infrastructure like, presence of water, enough health care provider, number of delivery room, number of waiting area, and the like in generally , favorable delivery room means satisfactory room for performing pain management practice and attending delivery

**Question 3.** Under Abstract, please correct the Spelling

**Response 3:** We amend it and corrected it accordingly

**Question 4. Under data quality control** please make it brief and clear, and put it under the analyses part, not separate section needed. However, short and clearer...

**Response 4:** We amend it and corrected it accordingly. The data quality control was written under data analyses and data processing section thanks for your nice comment

**Question 5. Under table 2 replace the word variables by** characteristic

**Response 5:** It was corrected according your suggestion

**Question 6.** Delete both figure 1 and figure two

Response 6. It was corrected accordingly

**Question 7**. What is the main dependent variable? Any non-pharmacological practice use. On the other hand, you asked so many non-pharma methods. What is your dependent variable here?

**Response 7.** The main dependent variable/ outcome variable was practice

**Question 8.** If it is no significant, take them out of the table,

Too many table and too many findings. Make it hard to follow.

**Response 8.** Under the last table correction was made according to you nice comment and suggestion

**Question 9. S**separate the sentences. One sentence one paragraph not pragmatically appropriate. In addition, even merge it with following or any other proper -related topics in another paragraph

Response 9. In discussion section, correction was made accordingly based on both reviewers comment and suggestion

**Question 10.** How it is line with.... interpret give suggestion, give your comment .. What other are going to do with this knowledge. Comment with your recommendation related to these findings

**Response 10: Correction was made thanks in depth, for your nice comment**

**Point by point response to Reviewer# 2**

**Question 1.** Native speaker should edit manuscript.
There are too many grammatical errors in the manuscript. Please correct!
There are many spelling mistakes in the manuscript. Please correct!
**Response 1:** We would like to say thank you very much for your invaluable comments and suggestions. We considered and modified and rewrote again based on your constructive issues regarding language, coherence and comprehensibility of the manuscript

**Question 2:** Is religion of the health professional is effect on good or poor practice of labour pain management? Please briefly comment with a sentence!
Two yes are written in the last line in table 3 (on Acupuncture). One of them should be no

**Response 2: religion have no effect on the pain management practice but the main reason why we write it is to know the distribution of health care providers in the point of religion** Thanks, a lot .

**Question3:** The discussion was written very poorly. This section should be improved with new references. It should be emphasized the new perspectives in the discussion section in the light of your results

**Response 3**: Thank you very much for providing additional reference and providing nice comment on discussion. We clarified in detail in discussion and conclusion section and we have used additional reference both reference 23 and 24 are additional based on your nice comment and suggestions

**Question 4:** modify the conclusion section.

**Response 4**: Thank you in depth. We amend it and conclusion was modified acceding to the result/ finding

Question 5. Two yes are written in the last line in table 3 (on Acupuncture). One of them should be no

Response 5. Thanks, we made correction
