# Supplementary material for: Non-pharmacological labor pain management practice and associated factors among skilled attendants working in public health facilities in Gamo and Gofa zone, Southern Ethiopia: A cross-sectional study
Source: PLoS One. 2022 Apr 21;17(4):e0266322. doi: 10.1371/journal.pone.0266322 (PMC9022872; doi:10.1371/journal.pone.0266322)
Supplement: Attachment — Submitted filename: Response to reviwers biresaw.docx [file pone.0266322.s004.docx]

**Author’s Point-by-Point Response to the Reviewer's and Editors Reports**

**Title**: Non-Pharmacological Labor Pain Management Practice and Associated Factors among Skilled Attendants working in public health facilities in Gamo and Gofa zone, Southern Ethiopia: A cross-sectional study

**Corresponding author**: Biresaw Wassihun /[bireswas@gmail.com](mailto:bireswas@gmail.com)

**Authors**

**1. Biresaw Wassihun**

2. Yosef Alemayehu

**Manscurpuit number:** PONE-D-20-00957

**Journal**: **PloS one**

**Article type:** Research article

**Point by point response to Reviewers and Editors**

First of all, the authors would like to thank Plos one Journal editors and the respective reviewers for reviewing our manuscript and providing the necessary comments to be corrected. As per the comments given, we have made corrections point by point to comment. The authors tried to answer all the issues raised by the editorial team and reviewers. Thank you again providing this interesting and constrictive comment

**Point by point response to Editor**

**Dear** Nülüfer Erbil, Ph.D., Prof

1. Please ensure that your manuscript meets PLOS ONE's style requirements

**Response:** Thank you very much we had applied journal requirement

2. Thank you for submitting your manuscript to PLOS ONE. After careful consideration, we feel that it has merit but does not fully meet PLOS ONE’s publication criteria as it currently stands. Therefore, we invite you to submit a revised version of the manuscript that addresses the points raised during the review process3. Please amend either the title on the online submission form (via Edit Submission) or the title in the manuscript so that they are identical

**Response:** Dear respected Editor Nülüfer Erbil, Ph.D., Prof thank you for providing interesting and constrictive comments so, we will provide each separately, thank you again

**Point by point response to Reviewer# 3**

**Reviewer #2:** The authors mad the corrections are given. This study can raise awareness about good practices, especially in developing countries. For me, ıt is suitable for publication.

**Response:** We would like to say thank you very much for your invaluable comments and suggestions. We considered and modified and rewrote again based on your constructive issues regarding language, coherence, and comprehensibility of the manuscript

**Reviewer #3**: The manuscript has been improved much. However, I do have some major comments.
**Question 1**. There are still many grammar and spelling errors in the article. For example, “Associated factors among skilled” in the abstract, the first letter should be lower case. “in Southern, Ethiopia”, please add a period at the end of the sentence. Same errors exist in other parts of the manuscript, please carefully check the manuscript and correct these errors

**Response 1:** We would like to say thank you very much for your invaluable comments and suggestions. We considered, modified, and rewrote again based on your constructive issues regarding language, and grammar. The abstract was corrected as follows

The health and well-being of a mother and child at birth largely determine the future health and wellness of the entire family. Labor pain management is crucial to ensure the quality of obstetric care but it is one of the neglected areas in obstetrics. Therefore, this study aimed to assess the practice of labor pain management and associated factors among skilled attendants working in public health facilities in Southern, Ethiopia from November 1–January 26, 2019

**Question 2.** Introduction section, the authors introduced “labor pain management” and its role in the first paragraph. However, the transition to maternal mortality in the second paragraph seems illogical. “Labor pain management” is not a key factor of maternal mortality

**Response 2:** Thank you very much. Dear respected reviewer as you know that one of the factors that affect delivery in public health institutions is the lack of health care providers' support and inadequate quality that is related to pain relief. Based on literature Lack of labor pain management practice is one of the unspoken causes of low utilization of institutional delivery so if there is low utilization of institutional delivery there is a high number of home delivery. Home delivery itself as its contribution to maternal mortality and in Ethiopia majority 74% of mothers gave birth in the home so lack of continuous labor support or lack of compassionate and respectful maternity care will be one of the indirect causes of maternal mortality and morbidity.

Finally, thank you again and we will as modify based on your comment and suggestion

**Question 3.** In the methods section, the content is too much and should be reduced. There are some improper English expressions. This research is indeed a facility-based investigation, no need to describe too much the “study area and period” or population

**Response 3:** It was corrected according to your suggestion, as follows

The study was conducted in selected public health facilities in Gamo and Gofa zone in south Ethiopia, from November 1–January 26, 2019

The source population study area and period was corrected accordingly thank you very much

**Question 4**. Results section, just summarize the key findings, 3 or four tables are enough.

**Response 4.** It was corrected according to your suggestion and the number of tables was reduced

**Question 5** . Does the score vary by facilities or by regions (Gamo vs Gofa)?

**Response5** : As a suggestion, we will consider it in the future to assess the disparity of using non-pharmacological labor pain management between health facilities taking health facility as one of the independent variable even including other private health facility

Thanks for your advance

**Question 6.** In the discussion section, besides discussing the status and associated factors of “non-pharmacological labor pain management” among skilled attendants in public health facilities, the authors should address how to improve the practice based on current findings and the guide released by the government.

**Response 6. The correction was made thanks in-depth, for your nice comment**

The identified factors should be addressed at both institutional and national levels to increase pain management practice for expectant mothers. Providing training for health care providers on non-pharmacological labor pain management and crating satisfactory delivery room is vital to increases practice of labor pain management. In addition, it is important for maternity care providers to respect the individuality of each client and be prepared to modify the delivery environment to attract more mothers to the health facility

Thanks for your nice comment
