# Supplementary material for: Non-pharmacological labor pain management practice and associated factors among skilled attendants working in public health facilities in Gamo and Gofa zone, Southern Ethiopia: A cross-sectional study
Source: PLoS One. 2022 Apr 21;17(4):e0266322. doi: 10.1371/journal.pone.0266322 (PMC9022872; doi:10.1371/journal.pone.0266322)
Supplement: Attachment — Submitted filename: Response to reviwers biresaw.docx [file pone.0266322.s005.docx]

**Author’s Point-by-Point Response to the Reviewer's and Editors Reports**

**Title**: Non-Pharmacological Labor Pain Management Practice and Associated Factors among Skilled Attendants working in public health facilities in Gamo and Gofa zone, Southern Ethiopia: A cross-sectional study

**Corresponding author**: Biresaw Wassihun /[bireswas@gmail.com](mailto:bireswas@gmail.com)

**Authors**

**1. Biresaw Wassihun**

2. Yosef Alemayehu

3. Teklemariam Gultie

**Manscurpuit number:** PONE-D-20-00957

**Journal**: **PloS one**

**Article type:** Research article

**Point by point response to Reviewers and Editors**

First of all, the authors would like to thank Plos one Journal editors and the respective reviewers for reviewing our manuscript and providing the necessary comments to be corrected. As per the comments given, we have made corrections point by point to comment. The authors tried to answer all the issues raised by the editorial team and reviewers. Thank you again providing this interesting and constrictive comment

**Point by point response to Editor**

1. Please ensure that your manuscript meets PLOS ONE's style requirements

**Response:** Thank you very much we had applied journal requirement

2. Thank you for submitting your manuscript to PLOS ONE. After careful consideration, we feel that it has merit but does not fully meet PLOS ONE’s publication criteria as it currently stands. Therefore, we invite you to submit a revised version of the manuscript that addresses the points raised during the review process

**Response:** Dear respected Editor thank you for providing interesting and constrictive comments so, we will provide each separately, thank you again

**Point by point response to Reviewer# 4**

**Reviewer #4:**  Thank you for the opportunity to review this manuscript under title Non-Pharmacological Labor Pain Management Practice and Associated Factors among Skilled Attendants working in public health facilities in Gamo and Gofa zone, Southern Ethiopia: A cross-sectional study.
The manuscript has been improved so much. However, I do have some minor comments.
There are still many grammar and spelling and style errors in the manuscript..

**Response:** We would like to say thank you very much for your invaluable comments and suggestions. We considered and modified and rewrote again based on your constructive issues regarding language, coherence, and comprehensibility of the manuscript

**Question 1**. There are still many grammar and spelling errors in the article. For example” Abstract section, in the key words changing the word of Labour to labor its better to write the term in manuscript in the same style (unified).

**Response 1:** We would like to say thank you very much for your invaluable comments and suggestions. We considered, modified, and rewrote again based on your constructive issues regarding language, and grammar. The abstract was corrected

**Question 2.** Introduction section, According to EDHS 2016 report MMR in Ethiopia was 412 per 100,000 live birth.The EDHS abbreviation of what? al so the MMR & FMoH in the paragraph two line 14.

**Response 2:** Thank you very much. Dear respected reviewer it was corected accordingly

**Question 3.** In the end of the introduction section put full stop point (.)

**Response 3:** It was corrected according to your suggestion

**Question 4**. Methods section, put full stop point (.) at the end of each paragraph for example:
A simple random sampling technique was used to select two hundred seventy-two (272) sampled
obstetric caregivers; (including midwives, nurses, and health officers) who were giving obstetric
care in the delivery room.

**Response 4.** It was corrected according to your suggestion

**Question 5** . Sample size and sampling technique, mention clearly the inclusion and exclusion criteria.
- In the Measurement part, Channing the word of Labour to labor in the line 6 also changing word of the cheek list to check list in the same line.

**Response5** : Thanks for your advance

**Question 6.** Results section, in general, revision the results interpretation
- Can chinning the (In this study, 272 health professionals) to (272 health professionals participated in the study, yielding a response rate of).

**Response 6. The correction was made thanks in-depth, for your nice comment**

**Question** 7 Discussion section, in general, need revision the discussion part
**Response7** : Thanks for your advance

**Question 8.** References section, check the number and the sequence of references in the text with the list of the references for example check the numbers 2, 5, 6, sequence

**Response8. we have made correction on it**

**Question 9** Sample size and sampling technique, mention clearly the inclusion and exclusion criteria

**Response9. we have made correction on it as followes**

**Inclusion criteria**

All obstetric caregivers; (including midwives, nurses, and health officers) who were giving obstetric care in the delivery room were inclideded in the study

**Exclusion criteria**

Those health care provider Absent during data collection due to [annual leave](https://uk.news.search.yahoo.com/search;_ylt=AwrIQZxM0ZJfS7IAEQB3Bwx.;_ylu=Y29sbwMEcG9zAzEEdnRpZAMEc2VjA3Nj?p=annual+leave&fr=yhs-Lkry-nt&fr2=p%3As%2Cv%3Aw%2Cm%3Anewsdd_bn_t%2Cct%3Abing&hspart=Lkry&hsimp=yhs-nt)
