# Supplementary material for: Non-pharmacological labor pain management practice and associated factors among skilled attendants working in public health facilities in Gamo and Gofa zone, Southern Ethiopia: A cross-sectional study
Source: PLoS One. 2022 Apr 21;17(4):e0266322. doi: 10.1371/journal.pone.0266322 (PMC9022872; doi:10.1371/journal.pone.0266322)
Supplement: Attachment — Submitted filename: Response to reviwers biresaw.docx [file pone.0266322.s006.docx]

**Author’s Point-by-Point Response to the Reviewer's and Editors Reports**

**Title**: Non-Pharmacological Labor Pain Management Practice and Associated Factors among Skilled Attendants working in public health facilities in Gamo and Gofa zone, Southern Ethiopia: A cross-sectional study

**Corresponding author**: Biresaw Wassihun /[bireswas@gmail.com](mailto:bireswas@gmail.com)

**Authors**

**1. Biresaw Wassihun**

2. Yosef Alemayehu

3. Teklemariam Gultie

**Manscurpuit number:** PONE-D-20-00957

**Journal**: **PloS one**

**Article type:** Research article

**Point by point response to Reviewers and Editors**

First of all, the authors would like to thank Plos one Journal editor, and the respective reviewers for reviewing our manuscript and providing the necessary comments to be corrected. As per the comments given, we have made corrections point by point to comment. The authors tried to answer all the issues raised by the editorial team and reviewers. Thank you again for providing this interesting and constrictive comment

**Point by point response to Editor**

1. Please ensure that your manuscript meets PLOS ONE's style requirements

**Response:** Thank you very much we had applied the journal requirement

2. Thank you for submitting your manuscript to PLOS ONE. After careful consideration, we feel that it has merit but does not fully meet PLOS ONE’s publication criteria as it currently stands. Therefore, we invite you to submit a revised version of the manuscript that addresses the points raised during the review process

**Response:** Dear respected Editor thank you for providing interesting and constrictive comments so, we will provide each separately, thank you again

**Point by point response to Reviewer# 3**

**Reviewer #3:**  Thank you for the opportunity to review this manuscript under the title non-Pharmacological Labor Pain Management Practice and Associated Factors among Skilled Attendants working in public health facilities in Gamo and Gofa zone, Southern Ethiopia:

A cross-sectional study.
The manuscript has been improved so much. However, I do have some minor comments.
There are still many grammar and spelling and style errors in the manuscript.

**Response:** We would like to say thank you very much for your invaluable comments and suggestions. We considered and modified and rewrote again based on your constructive issues regarding the language, coherence, and comprehensibility of the manuscript

**Question 1**. Introduction section. Labor pain management practices include pharmacological and non-pharmacological methods. The author should address the current research status of non-pharmacological pain management when introducing the importance of labor pain management)

**Response 1:** We would like to say thank you very much for your invaluable comments and suggestions. We considered, modified, and rewrote again based on your constructive comments

**Question 2.** 2. P17 Table 4, “AOR(≥5 years of experience)=7.0(1.60-3.05)” and “AOR(midwife)=7.13(1.06-4.77)”, I am wondering why the confidence intervals did not include the point estimates. The authors should double check the data and verify the results.

**Response 2:** Thank you very much. Dear respected reviewer, it was corrected accordingly

**Question 3.** In the results section, logistic analysis showed that knowledge was not statistically associated with practice. But in the discussion part, the authors ascribed differences in the practice between this study and others to the differences in knowledge. It is reasonable according to the common sense of KAP theory，but it is not supported by the results of this study. The authors should address it carefully and describe it more appropriately

**Response 3:** It was corrected according

**Question 4**. here are still many grammar and spelling errors in the manuscript.
(1) The introduction part, paragraph 2, line 9, “......facilitate mothers' ability for attachment and infant car giving”. It could be “infant care giving

**Response 4.** It was corrected according to your suggestion

**Question 5** . In the “data processing and analysis”, line 7, the criteria for selecting variables into logistic regression is p < 0.2, while the corresponding description in the results part is < 0.25 (the third row below Table 3).

**Response5** : Thanks for your advance

**Question 6.** The title of the Table 2 was “knowledge of non-pharmacological labor pain management practice in Gamo and Gofa zone Public health facilities southern, Ethiopia”. However, the table contains pharmacological and non-pharmacological items. Please use a suitable title instead.

**Response 6. The correction was made thanks in-depth, for your nice comment**

**Question** 7 In the Table 4, please give the full spelling of “HCP” in the footnote.

**Response 7. we have made correction on it**
