# Supplementary material for: Non-pharmacological labor pain management practice and associated factors among skilled attendants working in public health facilities in Gamo and Gofa zone, Southern Ethiopia: A cross-sectional study
Source: PLoS One. 2022 Apr 21;17(4):e0266322. doi: 10.1371/journal.pone.0266322 (PMC9022872; doi:10.1371/journal.pone.0266322)
Supplement: Attachment — Submitted filename: Response to reviwers biresaw.docx [file pone.0266322.s007.docx]

**Author’s Point-by-Point Response to the Reviewer's and Editors Reports**

**Title**: Non-Pharmacological Labor Pain Management Practice and Associated Factors among Skilled Attendants working in public health facilities in Gamo and Gofa zone, Southern Ethiopia: A cross-sectional study

**Corresponding author**: Biresaw Wassihun /[bireswas@gmail.com](mailto:bireswas@gmail.com)

**Authors**

**1. Biresaw Wassihun**

2. Yosef Alemayehu

3. Teklemariam Gultie

**Manscurpuit number:** PONE-D-20-00957

**Journal**: **PloS one**

**Article type:** Research article

**Point by point response to Reviewers and Editors**

First of all, the authors would like to thank Plos one Journal editor, and the respective reviewers for reviewing our manuscript and providing the necessary comments to be corrected. As per the comments given, we have made corrections point by point to comment. The authors tried to answer all the issues raised by the editorial team and reviewers. Thank you again for providing this interesting and constrictive comment

**Point by point response to Editor**

1. Please ensure that your manuscript meets PLOS ONE's style requirements

**Response:** Thank you very much we had applied the journal requirement

2. Thank you for submitting your manuscript to PLOS ONE. After careful consideration, we feel that it has merit but does not fully meet PLOS ONE’s publication criteria as it currently stands. Therefore, we invite you to submit a revised version of the manuscript that addresses the points raised during the review process

**Response:** Dear respected Editor thank you for providing interesting and constrictive comments so, we will provide each separately, thank you again

**Point by point response to Reviewer# 3**

 Reviewer #3: The manuscript has been improved. However, I do have some minor comments.

.**Response:** We would like to say thank you very much for your invaluable comments and suggestions. We considered and modified and rewrote again based on your constructive issues regarding the language, coherence, and comprehensibility of the manuscript

**Question 1**  P9, abstract, conclusion, line 3: the mentioned factors were significant predictors, not a predictor

**Response 1:** We would like to say thank you very much for your invaluable comments and suggestions. We considered, modified, and rewrote again based on your constructive comments

**Question 2.** P11, measurement, line 6, “labor pain” , the first letter should be uppercased. Line 9, “ ...less than the mean value were considered to have good practice...”. It could be “poor practice”.

**Response 2:** Thank you very much. Dear respected reviewer, it was corrected accordingly

**Question 3**.  P17 Table 4. Please add a footnote for “*

**Response 3.** It was corrected according to your suggestion

**Question 4**. There are still some grammar or style errors in the manuscript. For example, many sentences lack a full stop ( “.”). The authors should carefully check the documents to ensure that they meet the journal's format requirements

**Response 4** : Thanks for your advance
