# Supplementary material for: Non-pharmacological labor pain management practice and associated factors among skilled attendants working in public health facilities in Gamo and Gofa zone, Southern Ethiopia: A cross-sectional study
Source: PLoS One. 2022 Apr 21;17(4):e0266322. doi: 10.1371/journal.pone.0266322 (PMC9022872; doi:10.1371/journal.pone.0266322)
Supplement: Attachment — Submitted filename: Response to reviwers.docx [file pone.0266322.s009.docx]

**Author’s Point-by-Point Response to the Reviewer's and Editors Reports**

**Title**: Non-Pharmacological Labor Pain Management Practice and Associated Factors among Skilled Attendants working in public health facilities in Gamo and Gofa zone, Southern Ethiopia: A cross-sectional study

**Corresponding author**: Biresaw Wassihun /[bireswas@gmail.com](mailto:bireswas@gmail.com)

**Manscurpuit number:** PONE-D-20-00957

**Journal**: **PloS one**

**Article type:** Research article

**Point by point response to Reviewers and Editors**

First of all, the authors would like to thank Plos one Journal editor and the respective reviewers for reviewing our manuscript and providing the necessary comments to be corrected. As per the comments given, we have made corrections point by point to comment. The authors tried to answer all the issues raised by the editorial team and reviewers. Thank you again for providing this interesting and constrictive comment

**Point by point response to Editor**

1. Please ensure that your manuscript meets PLOS ONE's style requirements

**Response:** Thank you very much we had applied the journal requirement

2. Thank you for submitting your manuscript to PLOS ONE. After careful consideration, we feel that it has merit but does not fully meet PLOS ONE’s publication criteria as it currently stands. Therefore, we invite you to submit a revised version of the manuscript that addresses the points raised during the review process

**Response:** Dear respected Editor thank you for providing interesting and constrictive comments so, we will provide each separately, thank you again

**Point by point response to Reviewer# 3**

 Reviewer #3: There are still some errors in the manuscript. The authors must ensure that the grammar, format, and punctuation meet the requirements of the journal..

.**Response:** We would like to say thank you very much for your invaluable comments and suggestions. We considered and modified and rewrote again based on your constructive issues regarding the language, coherence, and comprehensibility of the manuscript

**Question 1**  1.Abstract, Methods, “Institution-based cross-sectional study design was conducted from November 1– January 26, 2019”, It is better to be expressed as “An institution-based cross-sectional study was conducted

**Response 1:** Thank you very much. Dear respected reviewer, it was corrected accordingly

**Question 2** Abstract, Results, “37.5% (95%CI: 32, 43%) of health care providers……” , it should be “37.5% (95%CI: 32%, 43%). Please pay attention to that the AOR should be described as “(AOR=2.82, 95%CI: 1.56, 5.07)”

.**Response 2.** It was corrected according to your suggestion as follows

Overall, 37.5% (95%CI: 32%, 43%) of health care providers had a good practice on non-pharmacological labor pain management. Clinical experience of 5 years and above (AOR=2.91, 95%CI: 1.60, 5.42), favorable attitude (AOR=2.82, 95%CI: 1.56, 5.07), midwife profession (AOR=1.45, 95%CI: 1.98, 4.27), and working in satisfactory delivery rooms (AOR=3.45, 95%CI: 2.09, 7.43), were significantly associated with a health professional good practice of labor pain management

**Question 3**. Abstract, Conclusion, “It was observed that; having a favorable attitude, having ≥5 years of work experience, …...”. Please remove the “;” from the sentence.

**Response 3** : Thanks for your advance it “;” was removed

**Question 4.** Introduction, paragraph 2, line 7, “Studies have been shown that ......”, It should be “Studies have shown that.....”. Line 13, “Additionally, Anxiety, postpartum depression.......”, the first letter of “Anxiety” should be lower-case

**Response 4** : Thanks for your advance it was corrected accordingly

line 7. Studies have shown that

line 13, anxiety

**Question 5.** Methods, Exclusion criteria, “Those health care providers absent.....”, It should be “Those health care providers who were absent.....”.

**Response 5** : Thanks for your advance it was corrected accordingly as follows

Those health care providers who were absent during data collection due to [annual leave](https://uk.news.search.yahoo.com/search;_ylt=AwrIQZxM0ZJfS7IAEQB3Bwx.;_ylu=Y29sbwMEcG9zAzEEdnRpZAMEc2VjA3Nj?p=annual+leave&fr=yhs-Lkry-nt&fr2=p%3As%2Cv%3Aw%2Cm%3Anewsdd_bn_t%2Cct%3Abing&hspart=Lkry&hsimp=yhs-nt)

**Question 6:** Results, paragraph 1, line 2, The “SD” in “29.9 ± SD4.89” should be removed.

**Response 6** : Thanks for your advance it was corrected accordingly

**Question 7:** It is better to describe the results using past tense sentences.

**Response 7:** Thanks for your advance it was corrected accordingly

**Question 8:** Table 3, please use the percent sign correctly. And in the sentences that begin with “Health Care providers.....”, “Care” should be lower-case.

**Response 8**: Thanks for your advance it was corrected accordingly as followes

| S.no | List of cheek list | Observational cheek list to assess practice labor pain management | |
| --- | --- | --- | --- |
|  |  | **Yes No** | |
| 1. | Health care providers show the patient how to bear down during labor and delivery | 153(56.3%) | 119(43.7%) |
| 2. | Health care providers allow companion of her choice during labor and delivery | 154(56.6%) | 118(43.4%) |
| 3. | Health care providers massage the back to relieve labor pain | 210(77.2%) | 62(22.8%) |
| 4. | Health care providers use hot or cold pack compress to relieve labor pain | 182(66.9%) | 90(33.1%) |
| 5. | Health care providers use psychotherapy to relieve labor pain | 184(67.6%) | 88(32.4%) |
| 6. | Health care providers use relaxation/ breathing techniques to relieve labor pain | 182(66.9%) | 90(33.1%) |
| 7. | Health care provider allows the mother to ambulate/labor exercise | 158(58.1%) | 114(41.9%) |
| 8. | Health care providers use subcutaneous water injection | 2(0.7%) | 270(99.3%) |

**Question 9:** P16, Table 4, Please use correct table format. Row 5, collumn4, “1,87” should be “1.87”.

**Response 9: it was done accordingly thank you**
