# Supplementary material for: Non-pharmacological labor pain management practice and associated factors among skilled attendants working in public health facilities in Gamo and Gofa zone, Southern Ethiopia: A cross-sectional study
Source: PLoS One. 2022 Apr 21;17(4):e0266322. doi: 10.1371/journal.pone.0266322 (PMC9022872; doi:10.1371/journal.pone.0266322)
Supplement: Attachment — Submitted filename: Response to reviwers.docx [file pone.0266322.s010.docx]

**Author’s Point-by-Point Response to the Reviewer's and Editors Reports**

**Title**: Non-Pharmacological Labor Pain Management Practice and Associated Factors among Skilled Attendants working in public health facilities in Gamo and Gofa zone, Southern Ethiopia: A cross-sectional study

**Corresponding author**: Biresaw Wassihun /[bireswas@gmail.com](mailto:bireswas@gmail.com)

**Manscurpuit number:** PONE-D-20-00957

**Journal**: **PloS one**

**Article type:** Research article

**Point by point response to Reviewers and Editors**

First of all, the authors would like to thank Plos one Journal editor, and the respective reviewers for reviewing our manuscript and providing the necessary comments to be corrected. As per the comments given, we have made corrections point by point to comment. The authors tried to answer all the issues raised by the editorial team and reviewers. Thank you again for providing this interesting and constrictive comment

**Point by point response to Editor**

1. Please ensure that your manuscript meets PLOS ONE's style requirements

**Response:** Thank you very much we had applied the journal requirement

2. Thank you for submitting your manuscript to PLOS ONE. After careful consideration, we feel that it has merit but does not fully meet PLOS ONE’s publication criteria as it currently stands. Therefore, we invite you to submit a revised version of the manuscript that addresses the points raised during the review process

**Response:** Dear respected Editor thank you for providing interesting and constrictive comments so, we will provide each separately, thank you again

**Point by point response to Reviewer# 3**

 Reviewer #3:  The manuscript has been improved much, but it still needs further revisions by native English speakers.

.**Response:** We would like to say thank you very much for your invaluable comments and suggestions. We considered and modified and rewrote again based on your constructive issues regarding the language, coherence, and comprehensibility of the manuscript

**Question 1**   1.Abstract, Background.It seems better to remove the first sentence from this paragraph and delete “Therefore” in the third sentence.

**Response 1:** Thank you very much. Dear respected reviewer, it was corrected accordingly and the first sentence was removed and deleted see from the mother document

**Question 3** Abstract, Methods. The authors should state directly that this study is a KAP survey. Please make clear the relationship between institutional-based study and sampling technique, and rephrase this paragraph.

.**Response 2.** It was corrected according to your suggestion as follows

An Institution-based cross-sectional study design was conducted from November 1–January 26, 2019. A simple random sampling technique was used to select a total of 272 obstetric care providers. Data were collected using pretested, and structured questionnaires. Data were entered to Epi data version 3.1 statistical software and exported to SPSS 22 for analysis. Bivariate and multivariate logistic regression analyses were performed to identify associated factors. P-value <0.05 with 95% confidence level were used to declare statistical significance

**Question 3**. “Abstract, Conclusion. The first sentence, please clarify “.....the practice ......was poor in public health facilities in Gamo zone

**Response 3**: Thanks for your advance it “;” it was corrected as follows This study showed that the practice of non-pharmacological labor pain management was poor in public health facilities in Gamo and Gofa zone

**Question 4.** There are some inappropriate uses of punctuation marks or grammars in the manuscript. Please carefully check the manuscript. Here are some examples.
(1)Introduction, second paragraph, “Among the practice competencies stated;” , the semicolon could be a comma

Introduction, second paragraph, “However, its actual practice is not recognized. (15,16)”, delete the symbol “.”

**Response 4**: Thanks for your advance it was corrected accordingly

**Question 5.** Methods, “(272) sampled obstetric caregivers;”, “All obstetric caregivers;”, semicolons should be replaced with commas.

**Response 5**: Thanks for your advance it was corrected

**Question 6:** 4)Measurement, “Knowledge about Labor pain management methods was measured by a 14-item. Knowledge questionnaire adapted from previous studies”, It should be rephrased as “Knowledge about labor pain management methods was measured by a 14-item questionnaire adapted from previous studies”.

**Response 6**: Thanks for your advance it was corrected accordingly like Knowledge about labor pain management methods was measured by a 14-item questionnaire adapted from previous studies

**Question 7:** Measurement, “labor pain management practice was measured by 9- checklist items adapted from previous studies”, It should be rephrased as “Labor pain management practice was measured by 9-item checklist adapted from previous studies”.

**Response 7:** Thanks for your advance it was corrected accordingly Labor pain management practice was measured by a 9-item checklist adapted from previous studies

**Question 8:**  Discussion, the authors reused “the findings of this study” many times, it is better to use other expressions. There might be other grammar errors in the manuscript, the authors should carefully check the documents

**Response 8**: Thanks for your advance it was corrected accordingly
